# Supplementary material for: Benefits of using genomic insulators flanking transgenes to increase expression and avoid positional effects
Source: Sci Rep. 2019 Jun 11;9:8474. doi: 10.1038/s41598-019-44836-6 (PMC6560062; doi:10.1038/s41598-019-44836-6)

Benefits of using genomic insulators flanking transgenes to increase expression and avoid positional effects

Ana Pérez-González and Elena Caro*

Figure S1: Nucleotide sequences of MARs.

>Rb7
TCGATTAAAAATCCAATTATATTTGGTCTAATTTAGTTTGGTATTGAGTAAAACAAATTCGAAC
CAAACCAAAATATAAATATATAGTTTTTATATATATGCCTTTAAGACTTTTTATAGAATTTTCTTT
AAAAAATATCTAGAAATATTTGCGACTCTTCTGGCATGTAATATTTCGTTAAATATGAAGTGCT
CCATTTTTATTAACTTTAAATAATTGGTTGTACGATCACTTTCTTATCAAGTGTTACTAAAATGC
GTCAATCTCTTTGTTCTTCCATATTCATATGTCAAAATCTATCAAAATTCTTATATATCTTTTTCG
AATTTGAAGTGAAATTTCGATAATTTAAAATTAAATAGAACATATCATTATTTAGGTATCATAT
TGATTTTTATACTTAATTACTAAATTTGGTTAACTTTGAAAGTGTACATCAACGAAAAATTAGTC
AAACGACTAAAATAAATAAATATCATGTGTTATTAAGAAAATTCTCCTATAAGAATATTTTAAT
AGATCATATGTTTGTAAAAAAAAATTAATTTTTACTAACACATATATTTACTTATCAAAAATTTGA
CAAAGTAAGATTAAAATAATATTCATCTAACAAAAAAAAAAACCAGAAAATGCTGAAAACCCGG
CAAAACCGAACCAATCCAAACCGATATAGTTGGTTTGGTTTGATTTTGATATAAACCGAACCA
ACTCGGTCCATTTGCACCCCTAATCATAATAGCTTTAATATTTCAAGATATTATTAAGTTAACGT
TGTCAATATCCTGGAAATTTTGCAAAATGAATCAAGCCTATATGGCTGTAATATGAATTTAAAA
GCAGCTCGATGTGGTGGTAATATGTAATTTACTTGATTCTAAAAAAATATCCCAAGTATTAATA
ATTTCTGCTAGGAAGAAGGTTAGCTACGATTTACAGCAAAGCCAGAATACAAAGAACCATAAA
GTGATTGAAGCTCGAAATATACGAAGGAACAAATATTTTTAAAAAAATACGCAATGACTTGGA
ACAAAAGAAAGTGATATATTTTTTGTTCTTAAACAAGCATCCCCTCTAAAGAATGGCAGTTTTC
CTTTGCATGTAACTATTATGCTCCCTTCGTTACAAAAATTTTGGACTACTATTGGGAACTTCTTC
TGAAAATAGT

>chiMARs
GAATTCGGATCCGGAGGCTCAGAAAACGGCAGTTGGGCACTGCACTGCCCGGTGATGGTGCC
ACGGTGGCTCCTGCCACCTTCTTTGATATTCACTCTGTTGTATTTCATCTCTTCTTGCTGATGAA
AGGATATAACAGTCTGTGAGGAAATACTTGGTATTTCTTCTGATCAACGTTTTTGTAAGTAATG
TTGAATATTGGATAAGGCTGTGTGTCCTTTGTCTTGGGAGACAAAGCCCACAGCAGGTGGTG
GTTGGGGTGGTGGCAGCTCAGTGACAGGAGAGGTTTTTTTGCCTGTTTTTTTTTTTTTTTTAAG
TAAGGTGTTCTTTTTTCTTAGTAAAATTTCTACTGGACTGTATGTTTTGACAGGTCAGAAACATT
TCTTCAAAAGAAGAACCTTTTGGAAACTGTACAGCCCTTTTCTTTCATTCCCTTTTTGCTTTCTGT
GCCAATGCCTTTGGTTCTGATTGCGTTATGGAAAACGTTGATCGGAACTTGAGGTTTTTATTTA
TAGTGTGGCTTGAAAGCTTGGATAGCTGTTGTTACACGAGATACCTTATTAAGTTTAGGCCAG
CTTGATGCTTTATTTTTTCCCTTCGAAGTAGTGAGCGTTCTCTGGTTTTTTTCCTTTGAAACTGG
CGAGGCTTAGATTTTTCTAATGGGATTTTTTACCTGATGATCTAGTTGCATACCCAAATGCTTG
TAAATGTTTTCCTAGTTAACATGTTGATAACTTCGGATTTACATGTTGTATATACTTGTCATCTG
TGTTTCTAGTAAAAATATATGGCATTTATAGAAATACGTAATTCCTGATTTCCTTTTTTTTTTTTAT
CTCTATGCTCTGTGTGTACAGGTCAAACAGACTTCACTCCTATTTTTATTTATAGAATTTTATAT
GCAGTCTGTCGTTGGTTCTTGTGTTGTAAGGATACAGCCTTAAATTTCCTAGAGCGGTGCTCAA
TAAGGCGGGTTGTCACATGGGTTCAAATGTAAAACGGGTACGTTTGGCTGCTGCCTTCCCGAG
ATCCAGGACACTAAACTGCTTCTGCACTGAGGTATAAATCGCTTCAGATCCCAGGGAAGTGTA
GATCCACGTGCATATTCTTAAAGAAGAATGAATACTTTCTAAAATATTTTGGCATAGGAAGCA
AGCTGCATGGATTTGTTTGGGACTTAAATTATTTTGGTAACGGAGTGCATAGGTTTTAAACAC
AGTTGCAGCATGCTAACGAGTCACAGCGTTTATGCAGAAGTGATGCCTGGATGCCTGTTGCAG
CTGTTTACAGCACTGCCTTGCAGTGAGCATTGCAGATAGGGGTGGGGTGCTTTGTGTCGTGTT
CCCACACGTTGCCACACAGCCACCTCCCGGAACACATCTCACCTGCTGGGTACTTTTCAAACCA

TCTTAGCAGTAGTAGATGAGTTACTATGAAACAGAGAAGTTCCTCAGTTGGATATTCTCATGG
GATGTCTTTTTTCCCATGTTGGGCAAAGTATGATAAAGCATCTCTATTTGTAAATTATGCACTT
GTTAGTTCCTGAATCCTTTCTATAGCACCACTTATTGCAGCAGGTGTAGGCTCTGGTGTGCGCT

>TBS
TTCCTAACACCTGGAGAACCTTTTATGTACTTCACAACCCTCAATGCTGCTTCCAGGTGTGACT
GTTTGGGCTGCTGCATGAATTGACTCAGCACCTGCACTGCAAAGCTTATATCTGGCCTTGTGAT
TGTCAAGTAGAGAAGCTTTCCCACCAGTCTCTGATAGGATCCCACATCCTCTAACTCTGCATCA
TCAGTCTTTCCTAAGTGCTTGTCATATTCTACAGTAGTCAGCCTTTGGTTCTGTTCCATTGGGGT
TGACACTGGTCTGCAGCCTCCCAGACCAACACCTGATATCAGTTCCAGTGCATACTTCCTCTGG
TTCAGTAGGATTCCCTTTTCTGATCTCAGCACCTCAATGCCTAAGAAGTATTTTAGTTCTCCCAA
ATCTTTCATCTTGAAATGCTGATGCAGGGTTGCCTTTGCTTCTGAAATCAAAACATTGCTGCTG
CCTGCTATTAACAGATCATCCACGTAAATCAGGATTATGACAAGGTCAGTCCCCTCTCTTTTGG
TAAACAAGGAGTGATCATAAGCACTTTGCATAAAACCAGCCTGCATAAGGACAGTGGTAAGC
TTGATGTTCCACTGCCTTGATGCTTGCTTTAAACCATAGAGGGATTCAACAGCCTGCACACTTT
GGTCGCCCCTTGGCTGTGAAAACCCTGAGGCAGAGACATATAAACTTCTTCCATGAGGTCACC
TTGTAGAAAAGCATTGTTGACATCCATCTGGAAAAGGAACCAGCCTTTGGAAGCAGCAACAG
ATATGACAGCCTTTACAGTGACCATTTTGGCCACTGGAGAAAAGGTTTCATGGTAGTCAAGGC
CTTCTTGCTGAGTGTATCCCTTGGCCACTAGCCTTGCCTTAAACCTGTCAACTTCACCATTAGCT
TTGTATTTAATTTTGTACACCTATTTGGACCCTATAGGCTGTTTACCAGGGGGGTAAAGGGACA
ATCCCCCAGGTGTTATTATCCTCAAGAGCCTGTATCTCAAGGGACATAGCCTCCATCCATTTCT
CATCTTGGGCTGCTTCTTTGAAGGATCTAGGTTCAGTATAAGTGGAGAAAGCACTTAAATAAG
CTTGATAGTGAACAGGCAAGTGATCATAGGAAACATAGTTGGCTATAGGGTATGGGACATCC
CCAGAGCCTTTGTTTAGTGTCACAAAGTCCTTGAGCCAGATGGGAGGACCTGCATTGCGTTTA
GGTCTAGTGTGCAGGTTGGCTGGAACTGGAGAGGAATCAGCTACAGCAGGATGGTGCTCAA
ATTCAGCATTTGCTAGGTCAGGCTCAGCTGACTCAACTGACTCTGCAGGGGCATGCAGCCGGC
CTGAAGGTGCAGCTTCAGCTGAAATGGTATGATTTTGGGTAGAATGAGTCCCCAAAGTGATAT
CCTCATTGGCCTCTACATTGTCAGGAATAAGAACTGGAGTATCATCATCATCAGTATGAAAGCT
GGAAGGAAAAATATCATTATACACTAGCTGCAAGGCTGTATCTTCGGAGCTAAGTGAACCTGC
TTGAGTGAACATATCAGGTTCATGGGAGATGGACTCTTTGAAAGGGAAAGAATATGTGAGTG
ATTCATACACAGCATCTCTTTCCAATAGTATGACCGATCATTGTGGGTATAATGGTAGATGCCC
GGGACCAAGTCGATAGAAATTCAGTATTTTGCAACGAAAAACAGGACAGATTCAAGTTAAGTT
ATGAAATTCTCTGAAAACTACATCCCCGCTCACTATGATCACCTTATTATCCAAGTCATACAACC
TGTAACCCTTTTGAGTCTCAGAATAACCAATGAAGATGGTTTGTTTGGCTCTAGGTGCAAGTTT
GTCACCTTTGGGCAGTGTTGCTGCAAAAGCAAGACACCCAAACACTCTCAGATGATCAAGCTT
GGCATGTTTCTGATAGAGTAGTTCATATGGACATTTGCCTTGTAAGATTGGAGTAGGAAGTCT
ATTGATCAGGTATACAGCAGTCTTGACACAATCTCCCCAAAACCTGGTAGGTACACCACTCTG
AAACTTAAGTGCCCTTGCCATTTCAAGGATGTGTCTGTGCTTCCTCTCCACAACACCATTTTGTT
GTGGTGTGTAGGGACAGCTACTTTGATGAACAATCCCAAGAGAGGCCAGCAACTCATTACAA
CTT

>At S/MAR10
TGGCTATTGTTGTTATCATCAAAATCTATATTGATACTATTATACTCGATATGTTATAGACTTTA
TTAAAAAAAACTTTGTTTGTTTGCAGAAAGTGGATTTTTAATCAAATTTAATTGTGATGATTAC
AATGTCAATTGGTTATGCAAGATATTAATTAATATGTTTAAGATTTTTCACTTTTAATAAGTAAA
ATAAAATACGTCAAAAAAATTTCCTAAAGATAATGCCAAAAAGGTTAAATTATAGAAATTAAA
CTAAAATATATTAAAAGTTGACTTAAAAATGCAATGTTGACCACCGACCTAGGGGAAAACAAA

TTTACCGTATCTGGGATAGAATCTCTTCTGTATCATCTCAGATAAAGATTACTACATAAACACA
CTTTACAGTTTTACGACGCCCAAAATTCAATTCCCGGAGATTAAAAACCCTAAAACAAACACTG
GCGATTCGATTTTCCCTCCCAATTCAAATCATCTCTTAATCTTCTTCTTCTCACTATCCTCTCAAA
TTCGCCGCCGATCTTCCATCAATTTTGATTTCTCGCACCGAAATTTCGACTTTTCATCTCCTATCG
ACGAAACCCTAATTTTTCACACTCCAGAAATCGAACTTTGACGACGATGTTAATGGCTAAACCC

Figure S2: WGR data at the 21 mapped single insertion sites.

1

Control 1009

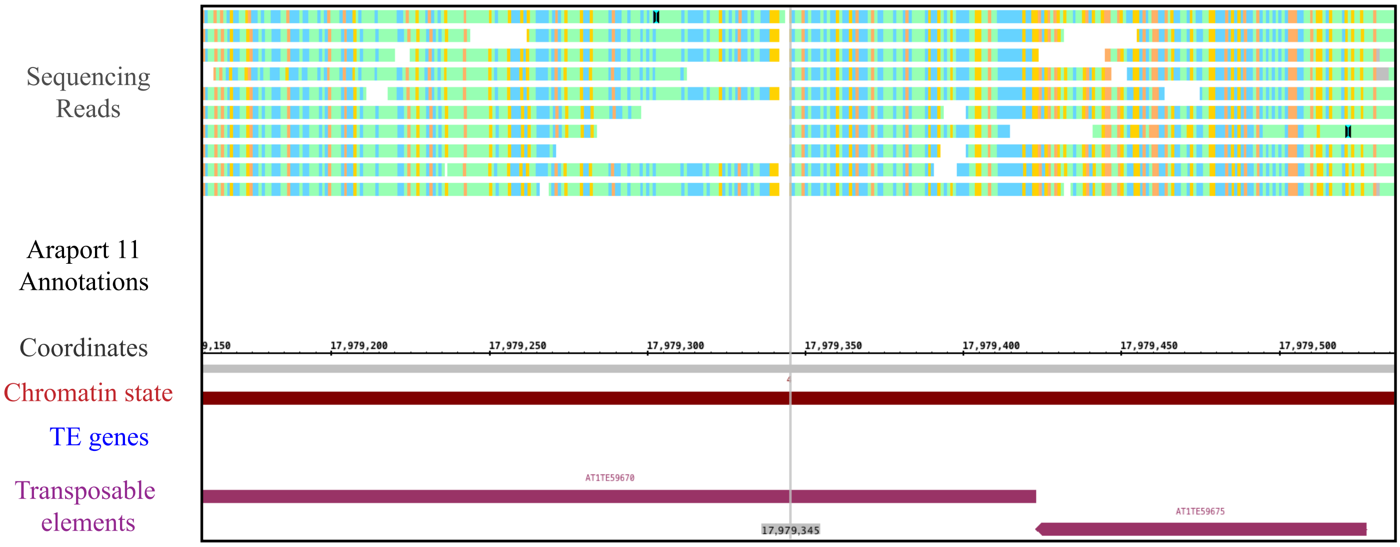

2

Control 1314

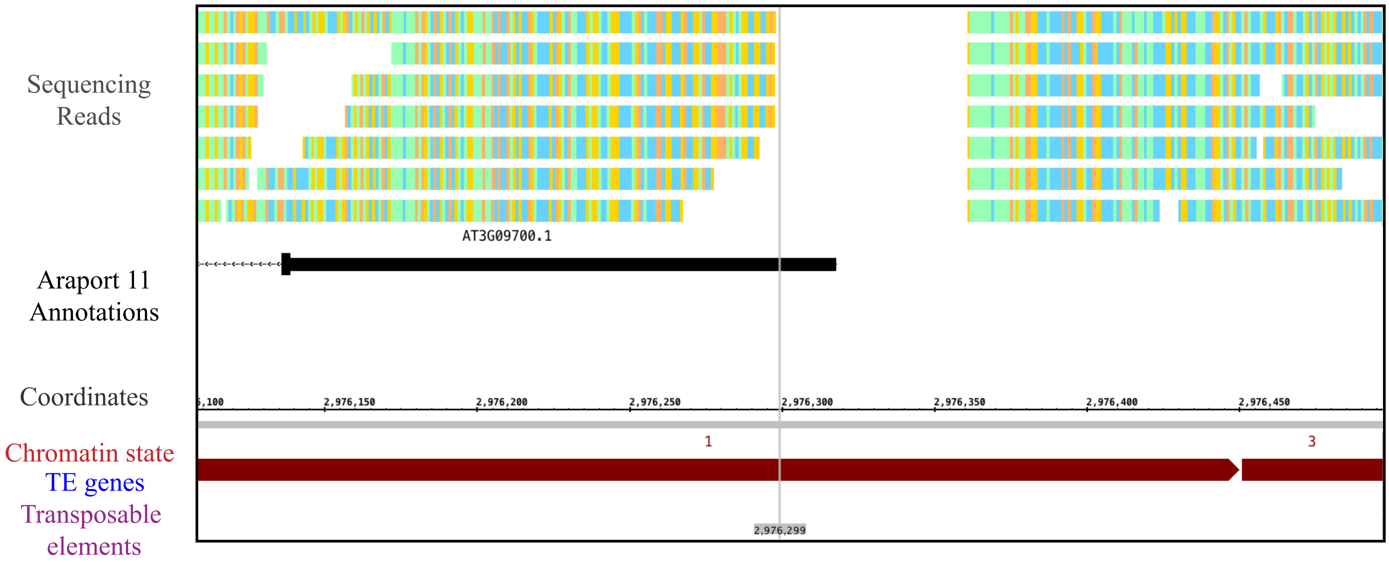

3

Control 1409

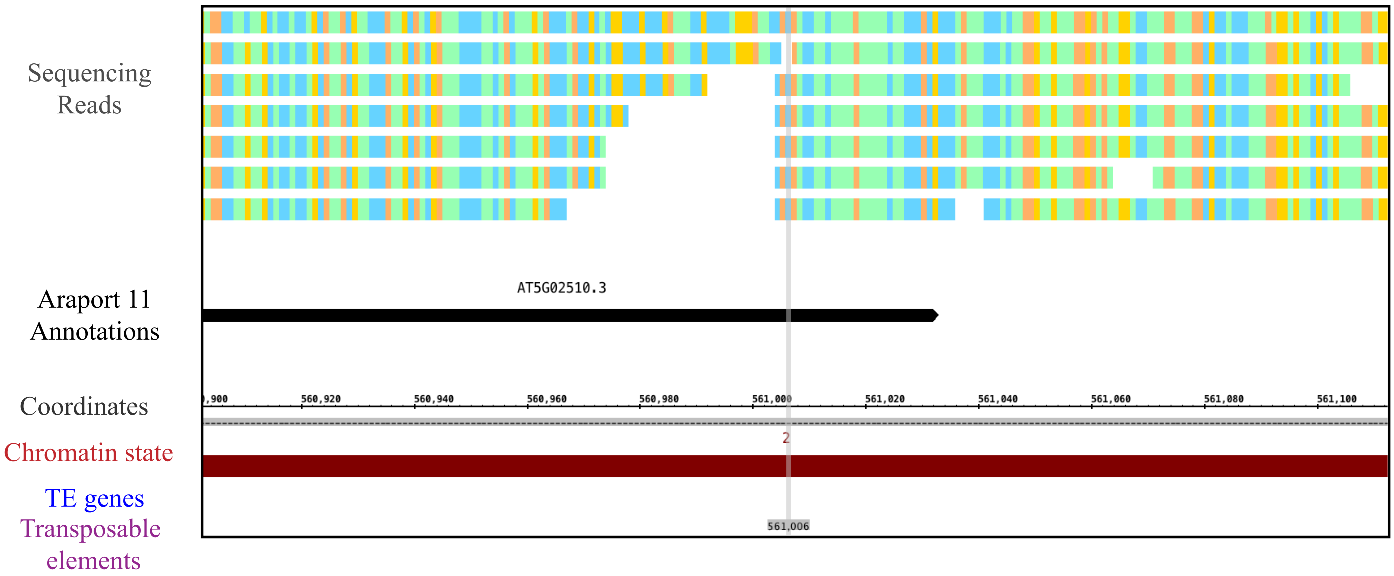

4

Control 3414

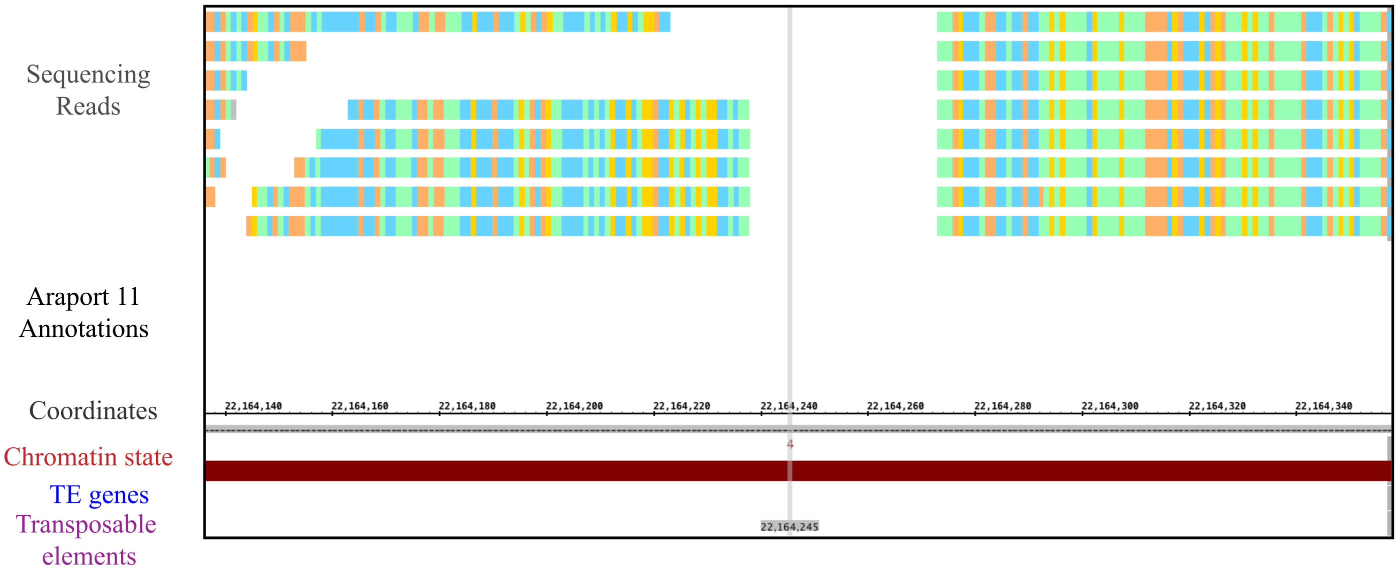

5

AtS/MAR10 0101

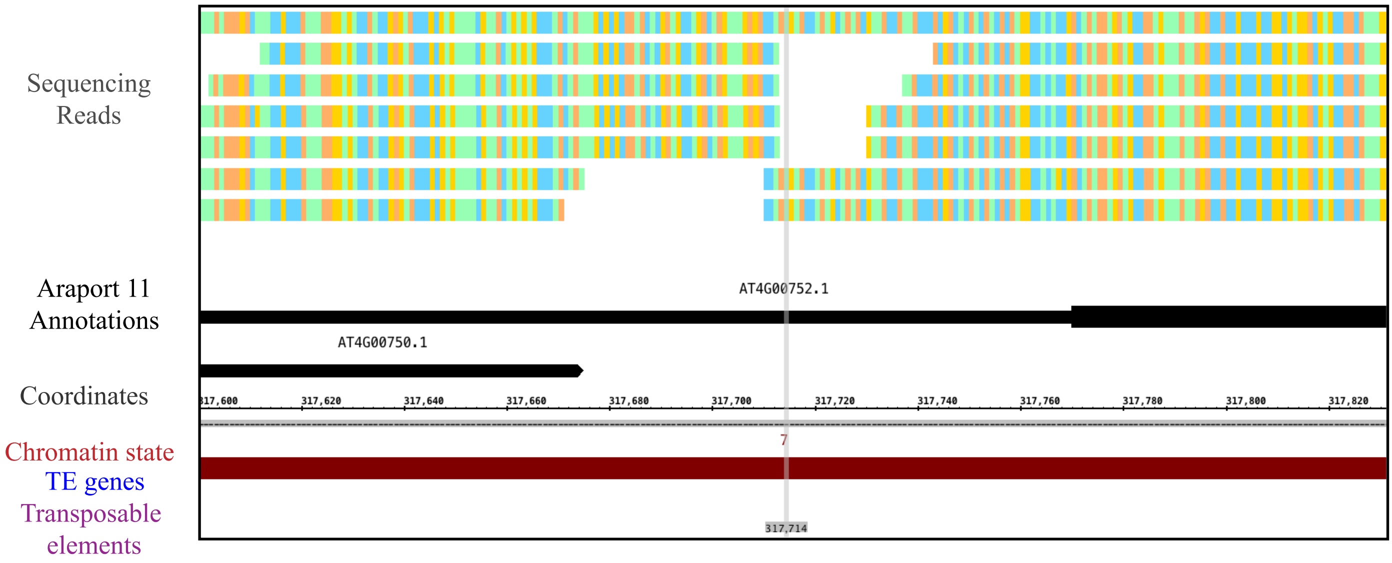

6

AtS/MAR10 1607

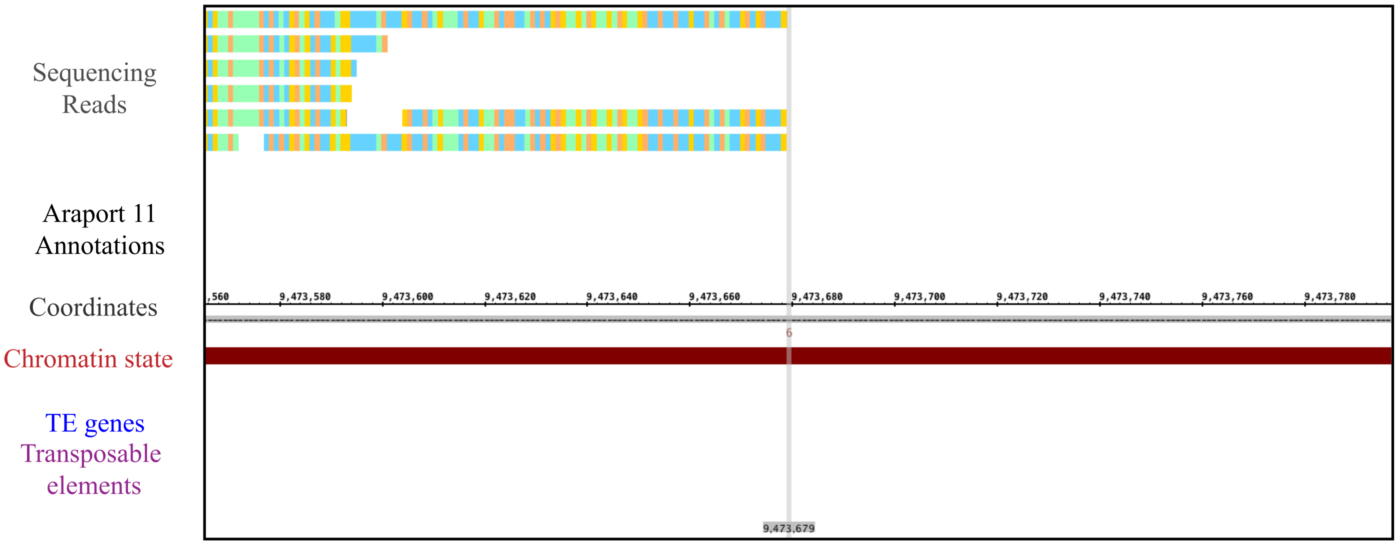

7

AtS/MAR10 2308

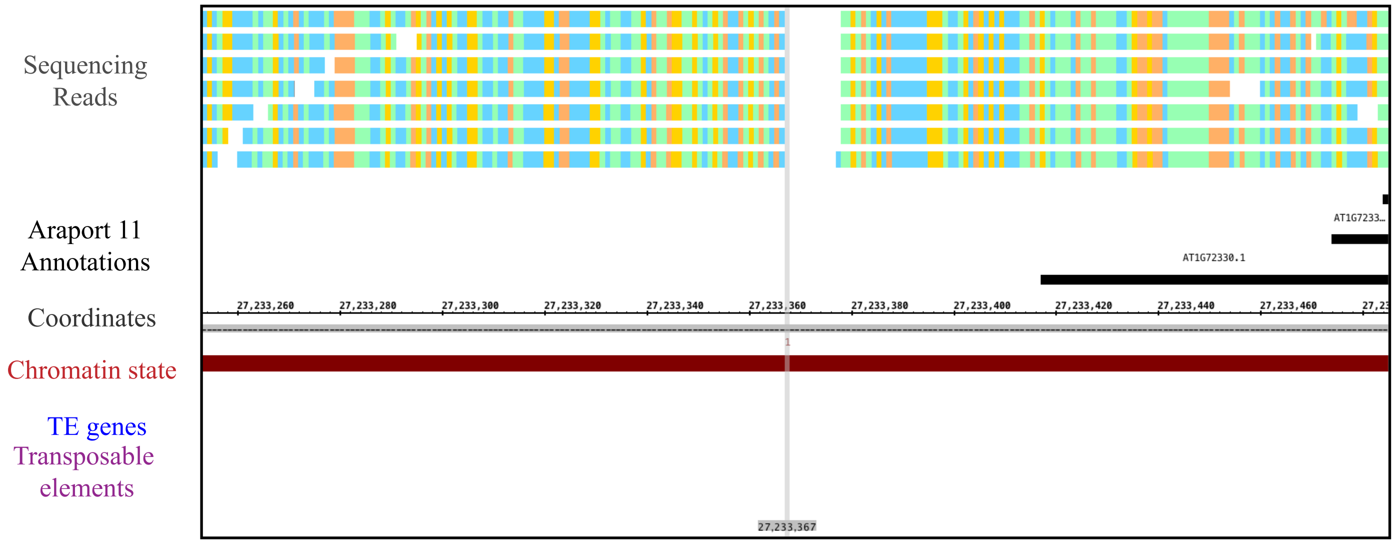

8

AtS/MAR10 4101

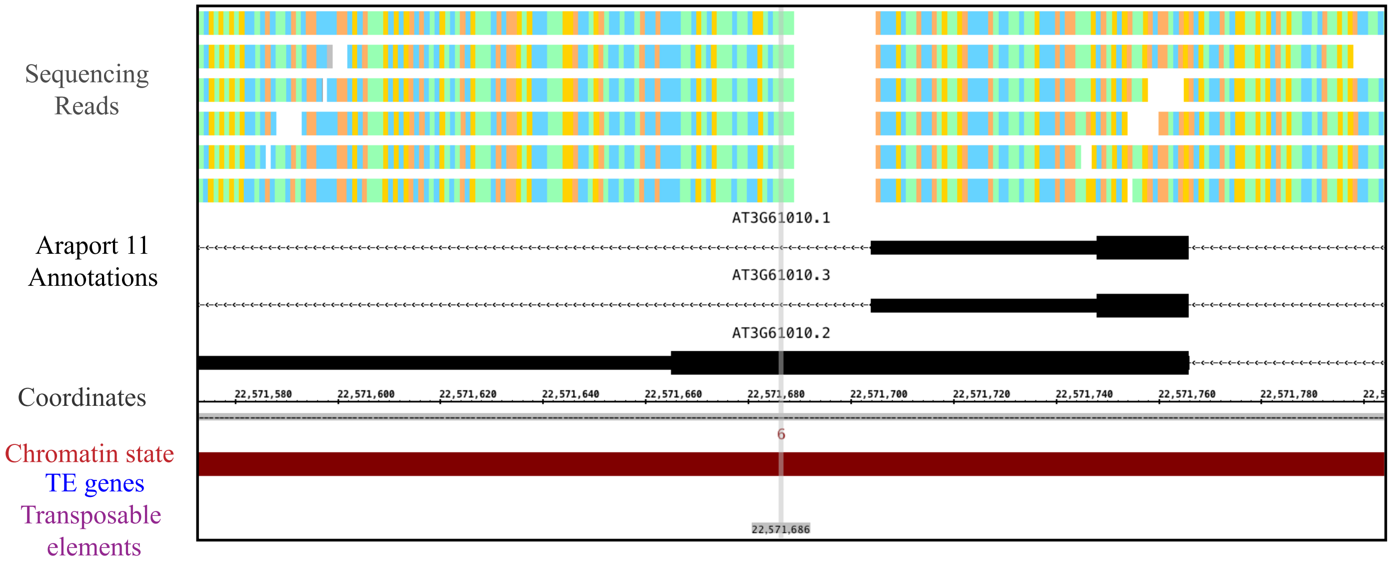

9

AtS/MAR10 4203

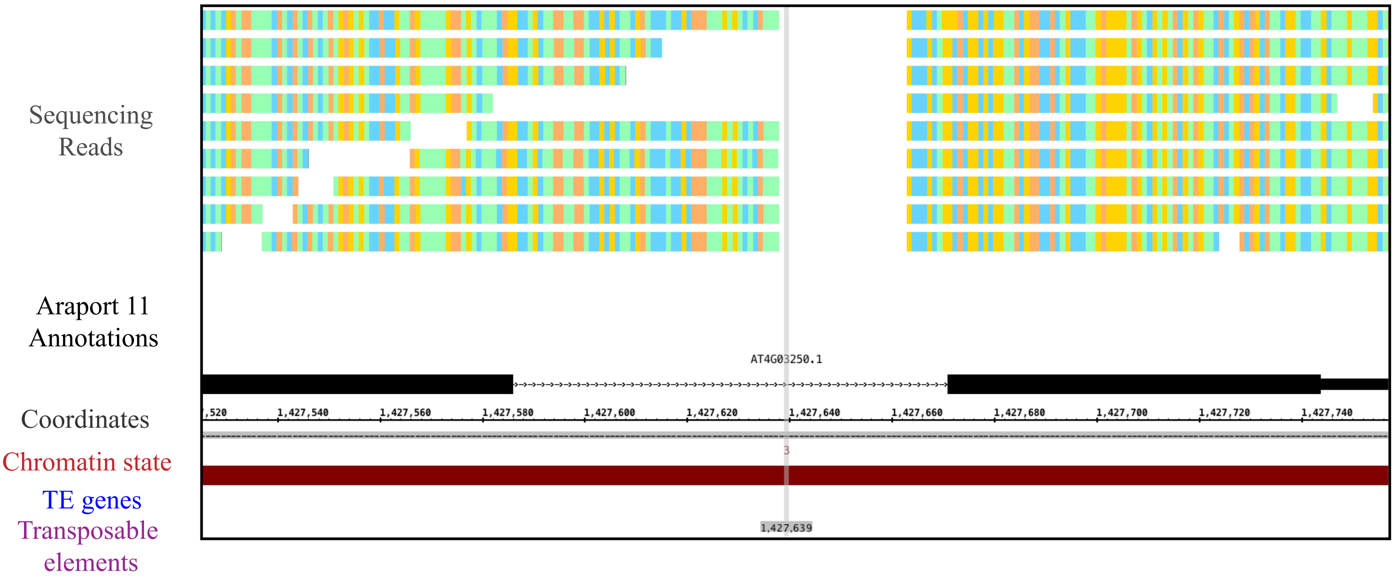

10

Rb7 0502

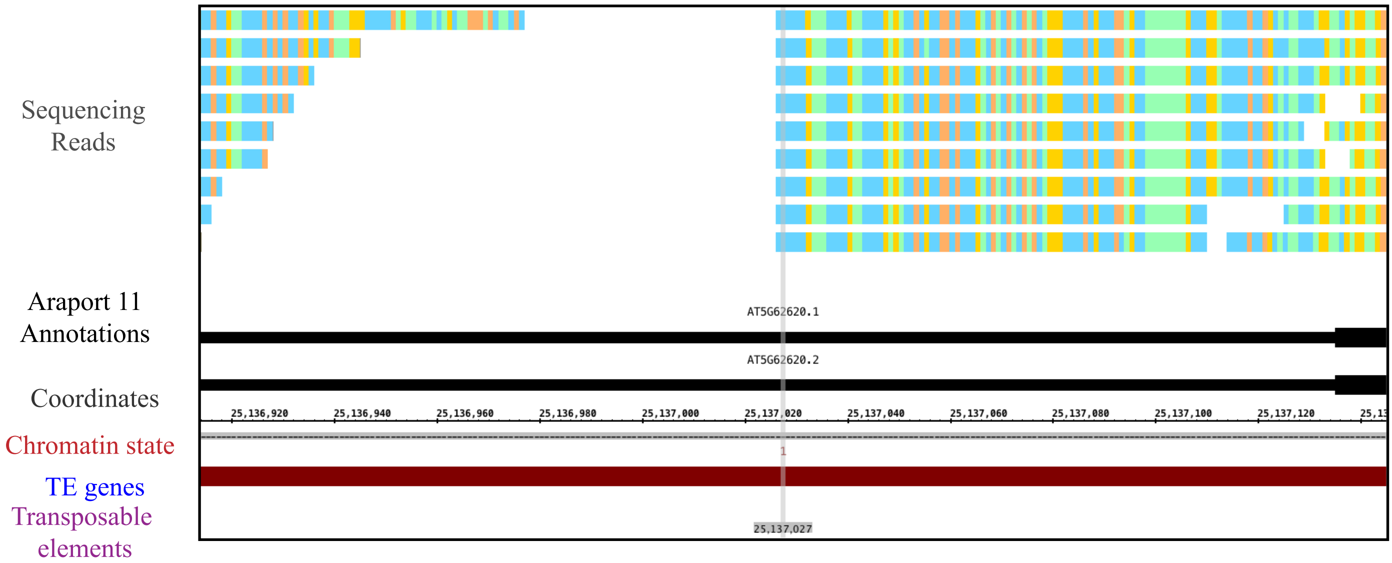

11

Rb7 0902

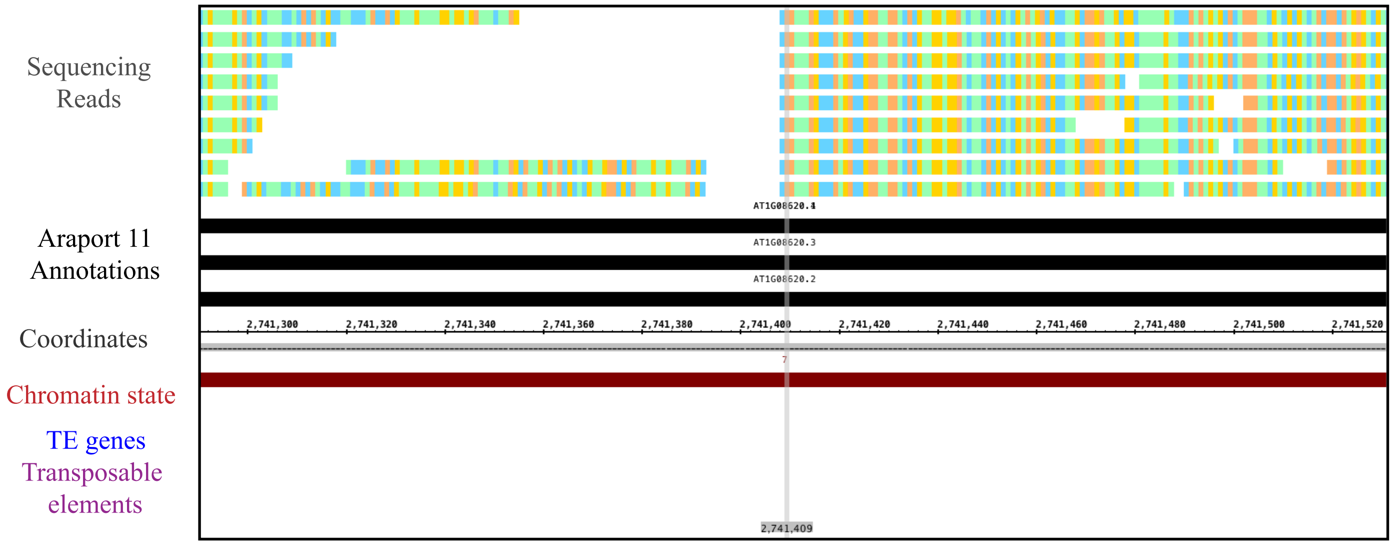

12

Rb7 1001

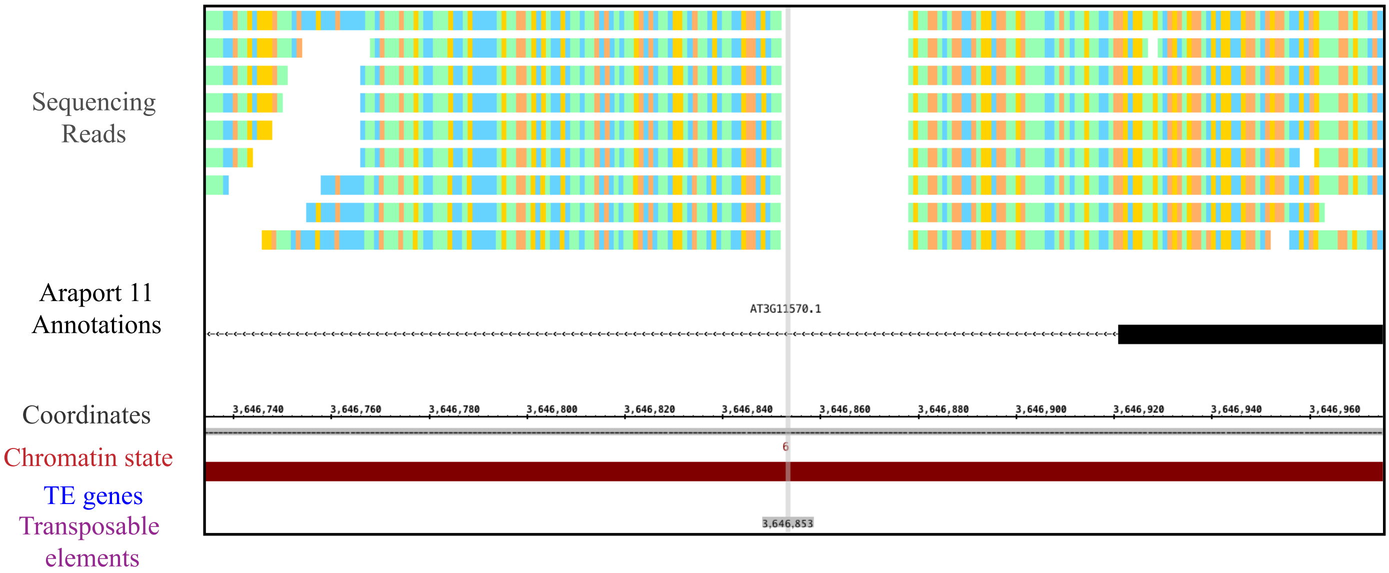

13

Rb7 2707

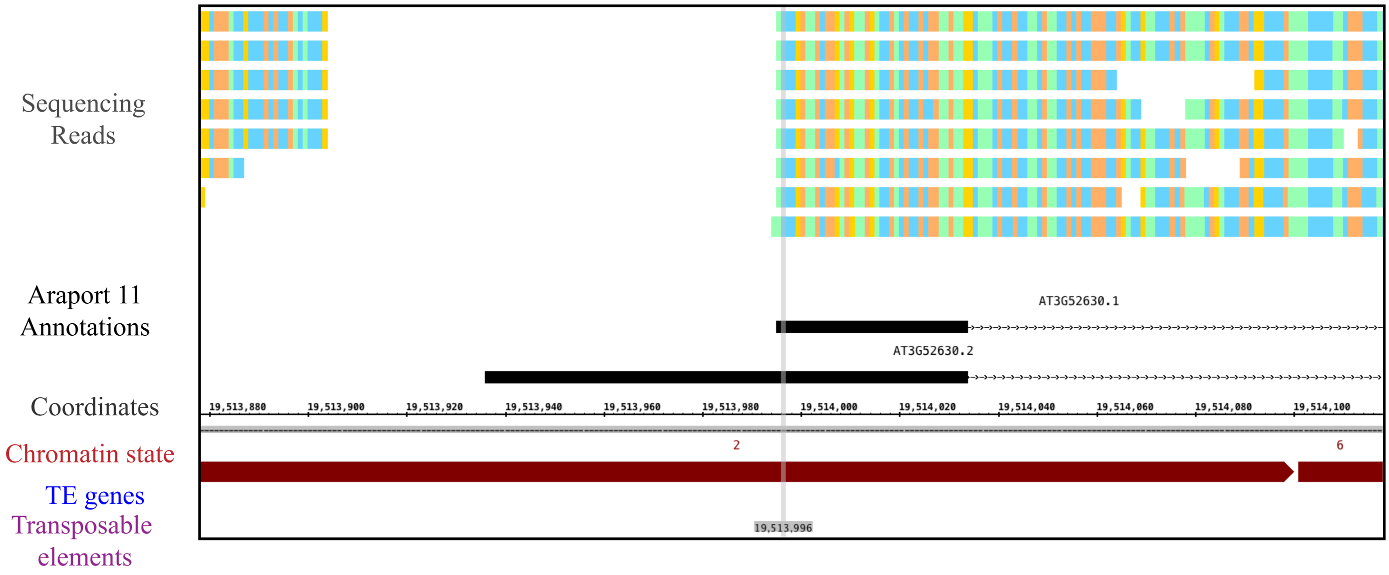

14

Rb7 4202

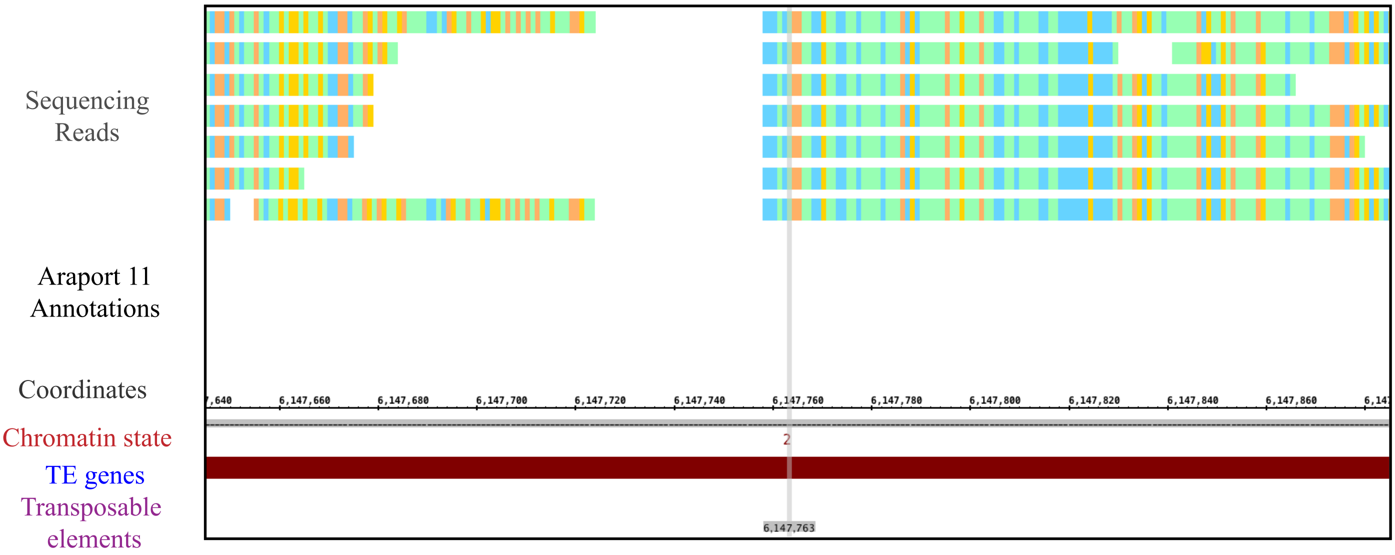

15

TBS0403

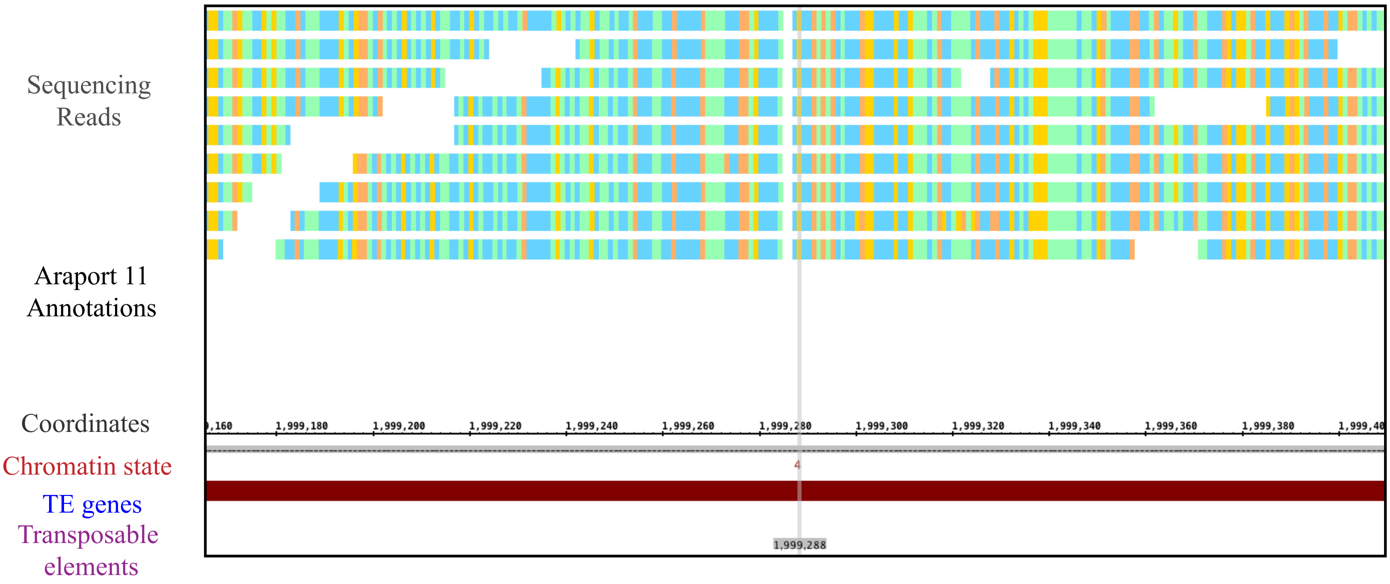

16

TBS 1109

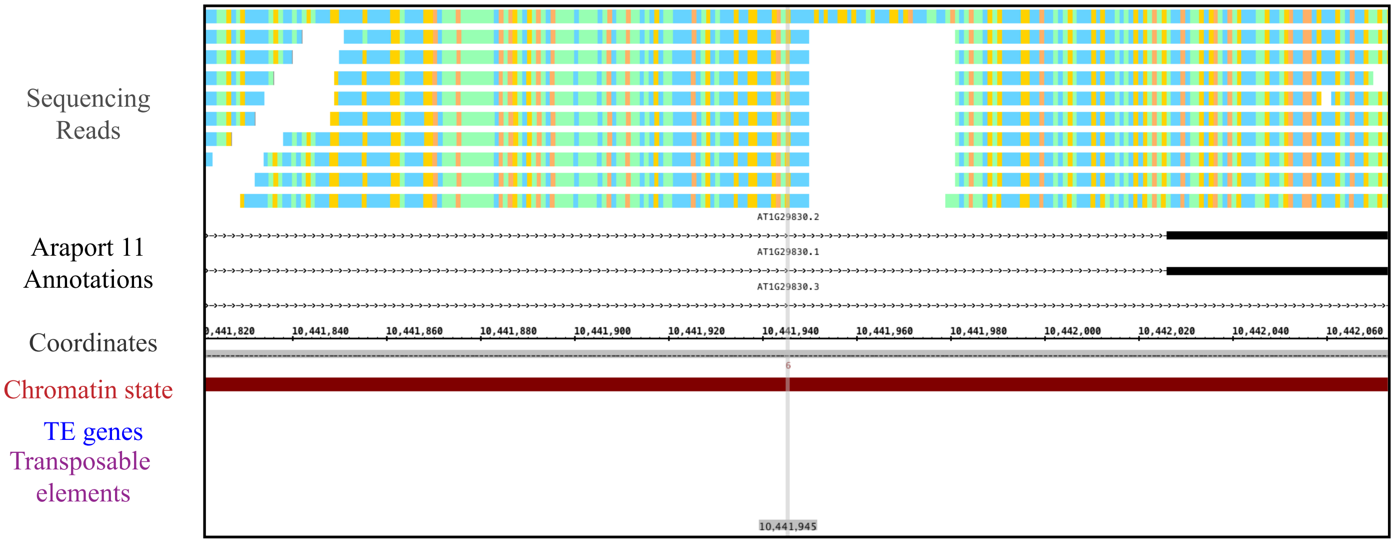

17

TBS 1503

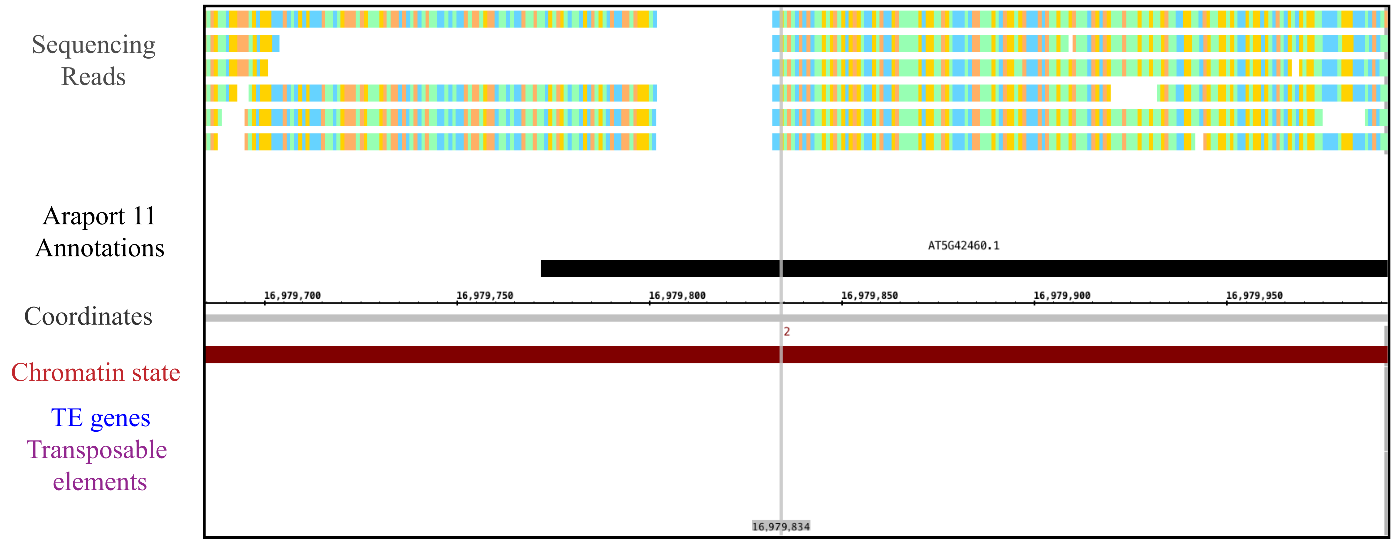

18

TBS 1806

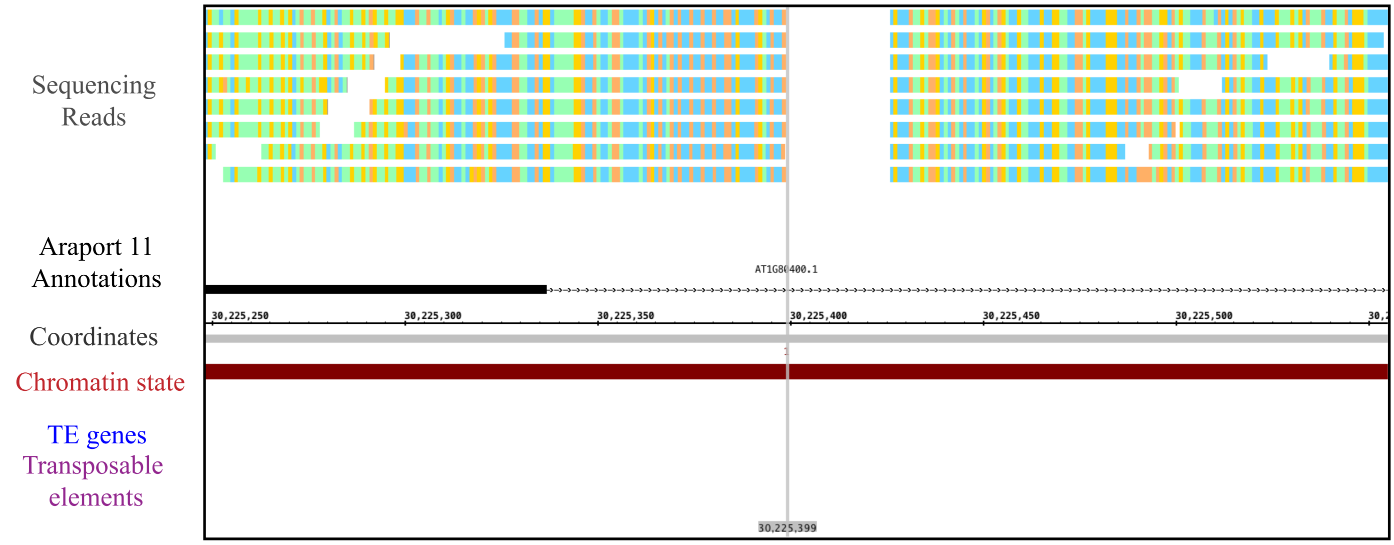

19

chiMARs 0112

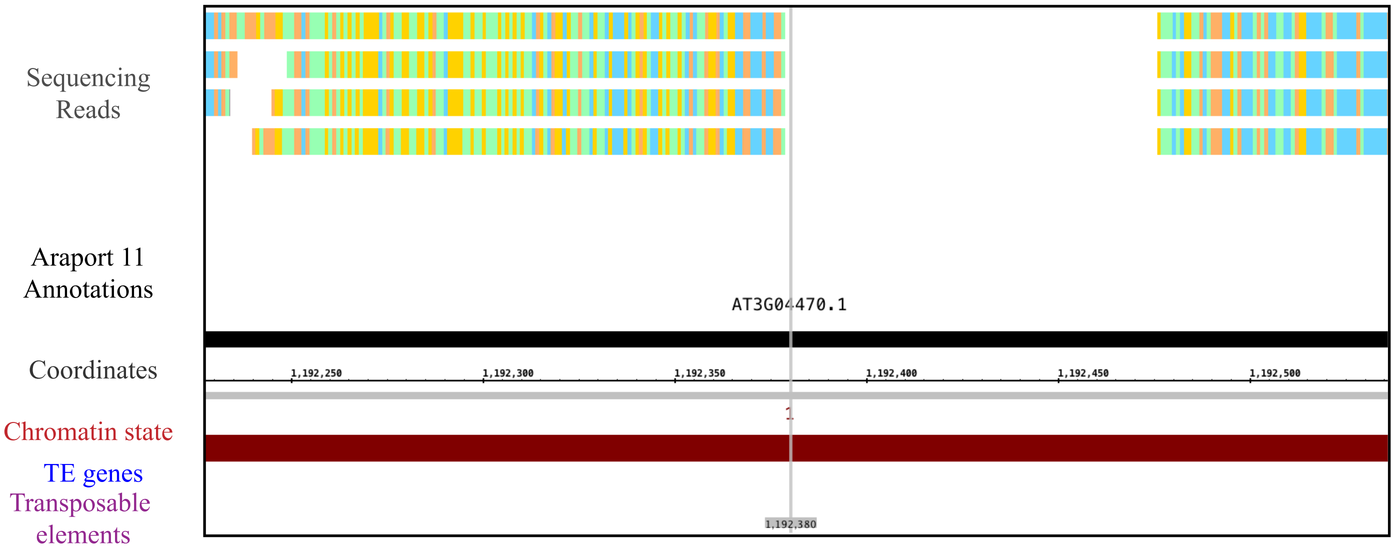

20

chiMARs 0613

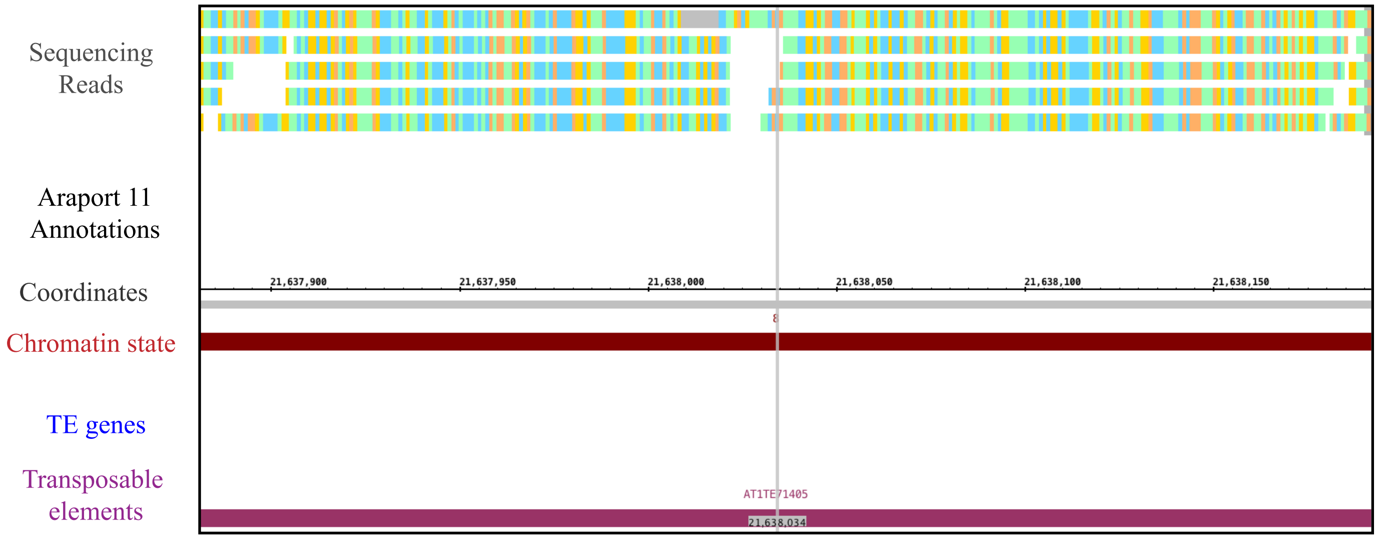

21

chiMARs 0711

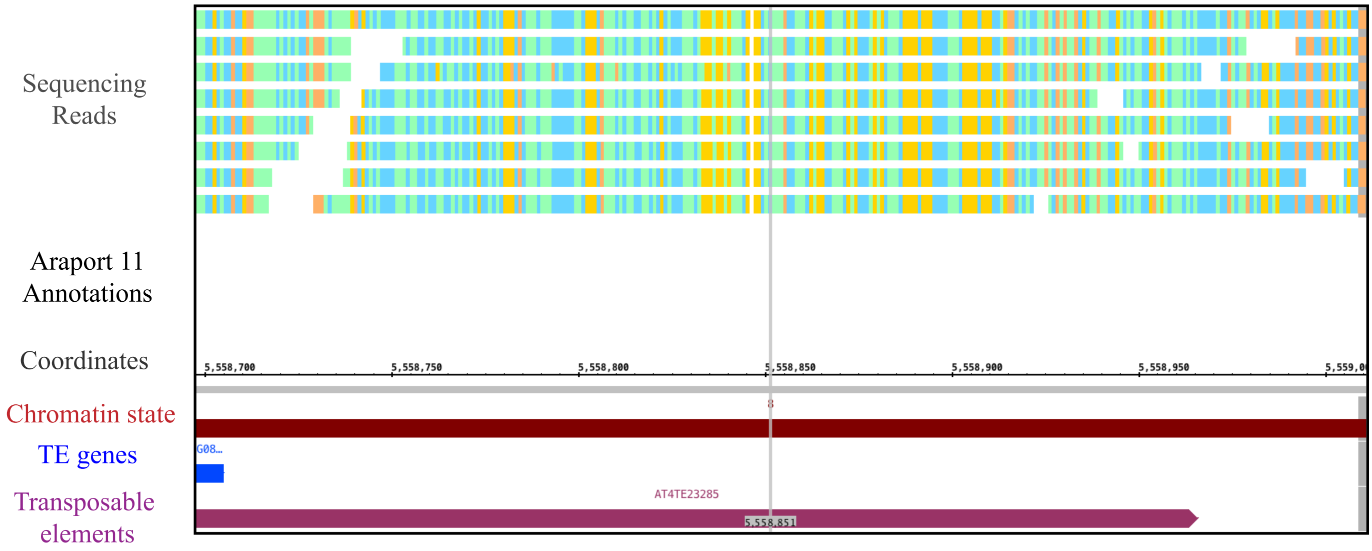

Supplement: Supplementary file 1 — Supplementary Figures S1 and S2 [file 41598_2019_44836_MOESM1_ESM.pdf]
